# Wisdom across interfaces: Adhesion mechanism and application potential of wet-adhesive hydrogel in periodontal tissue

**DOI:** 10.1016/j.bioactmat.2025.09.047

**Published:** 2025-10-14

**Authors:** Qinyu Duan, Xueyu Wang, Shan Wang, Zixin Yang, Weiwei Tan, Zhirui He, Shanshan Hu, Jinlin Song

**Affiliations:** a College of Stomatology, Chongqing Medical University, Chongqing, 401147, China; b Chongqing Key Laboratory of Oral Diseases and Biomedical Sciences, Chongqing, 401147, China; c Chongqing Municipal Key Laboratory of Oral Biomedical Engineering of Higher Education, Chongqing, 401147, China; d Chongqing Municipal Health Commission Key Laboratory of Oral Biomedical Engineering, Chongqing, 401147, China

**Keywords:** Periodontitis, Hydrogels, Wet adhesion, Microenvironment adaptation, Biological applications

## Abstract

Periodontitis, a chronic inflammatory disease of periodontal tissues closely linked to systemic health, presents significant therapeutic challenges due to the dynamic and moist microenvironment of these tissues. Current local therapeutic systems lack adaptability to this unique microenvironment, making it difficult to guarantee treatment efficacy at the target site. Wet-adhesive hydrogels, with their exceptional tissue adhesion and multifunctionality, demonstrate tremendous potential as emerging materials for periodontitis treatment. In this review, we systematically summarize recent advances in wet-adhesive hydrogels for periodontitis research, covering their adhesion challenges, mechanisms, material types, diverse biological applications and clinical translation. We begin with analyzing the unique anatomical structure and physiopathological microenvironment of periodontal tissues, revealing the fundamental causes that hinder long-term retention of local therapeutic systems. We then explore the chemical and physical adhesion mechanisms of wet-adhesive hydrogels, elucidating the scientific basis that supports their adaptation to the distinctive periodontal environment. Furthermore, we systematically categorize various adhesive hydrogel types along with their adjustable physicochemical properties, establishing a crucial link between adhesion mechanisms and clinical translation. We then summarized the multidimensional applications of wet-adhesive hydrogels in periodontitis, including wound protection and isolation, barrier membranes, stimuli-triggered drug delivery, hemostasis, electrical stimulation, stress conduction, and monitoring. Finally, we comprehensively discuss the current status and challenges of clinical translation. Our aim is to provide both theoretical foundation and practical guidance for developing next-generation intelligent and personalized periodontal treatment platforms.

## Highlights

• Analyze the adhesion challenges in wet periodontal microenvironment.
• Discuss adaptive wet-adhesion mechanisms for periodontal microenvironment.
• Classify wet-adhesive hydrogels by material sources.
• Summarize the applications of wet-adhesive hydrogels in periodontitis.
• Reveal clinical translation potential of wet-adhesive hydrogels.

## Graphical abstract

**Figure:**
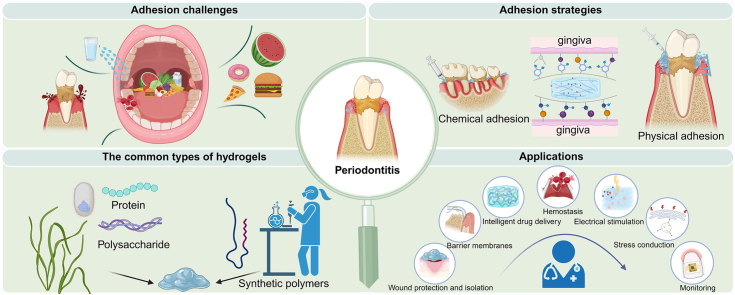

## 1 Introduction

Periodontitis is a chronic inflammatory disease initiated by dysbiotic dental plaque biofilm, characterized by progressive destruction of periodontal tissues, including the gingiva, periodontal ligament, cementum, and alveolar bone [1]. Clinically, affected patients exhibit gingival bleeding, periodontal pocket formation, alveolar bone resorption, and eventual tooth mobility or loss. Epidemiological surveys indicate that approximately 50% of adults globally suffer from varying severity of periodontitis, significantly impacting quality of life and the systemic health of patients, such as increasing the risk of systemic diseases including cardiovascular disease, diabetes, and chronic kidney disease [[2], [3], [4]]. For decades, mechanical debridement (subgingival scaling and root planing) combined with systemic medication has remained the primary treatment approach for periodontal diseases. While it temporarily controls infection, significant limitations persist [5]. In particular, the complex anatomical structure of periodontal pockets (deep pockets and furcation lesions) makes it difficult for mechanical debridement to completely remove biofilms, leading to high recurrence rates and the risk of causing bacteremia [6,7]. Furthermore, systemic antibiotic application is prone to induce drug resistance and systemic adverse effects [8]. These challenges have led to significant efforts to develop more effective local therapeutic strategies. Currently, various formulations such as ointments and mouthwashes have been adopted in clinical practice for periodontitis management [9,10]. However, existing local treatment systems face critical challenges in the complex oral environment. For instance, insufficient adhesion leads to rapid clearance by saliva, resulting in short retention time and low stability [11]. Furthermore, the current treatments’ single-function design fails to address the multidimensional therapeutic requirements of periodontitis, including infection control, inflammation suppression, tissue regeneration, and functional restoration.

In recent years, the rapid advancement of biomaterials has opened new avenues for periodontitis treatment. Hydrogels, as bioinspired functional materials, have demonstrated remarkable potential in achieving controlled and targeted drug release, improving pharmacokinetics, and enhancing drug bioavailability and selectivity. However, the unique oral environment—characterized by continuous endogenous saliva secretion, exogenous food/beverage washing, and physiological movements (e.g., mastication, speech, and swallowing)—endows periodontal tissues with highly moist and dynamic properties [12]. Conventional hydrogels often exhibit poor therapeutic effects due to their limited adaptability to wet environments and weak resistance to dynamic forces [13]. In contrast, microenvironment-adapted wet-adhesive hydrogels exhibit distinct therapeutic advantages for periodontal applications. Combining the inherent advantages of traditional hydrogels with robust interfacial adhesive capabilities, they form strong and durable adhesion to moist periodontal mucosa or root surfaces through synergistic chemophysical mechanisms. This enables them to effectively withstand salivary flushing and mechanical stress, thereby providing a stable platform for periodontitis therapy. Notably, their multifunctional integration capacity positions them as a pivotal technology to overcome the limitations of conventional treatments [14,15].

Over the past five years, several reviews have focused on hydrogel-based treatments for periodontitis. Yin et al. examined the pathophysiological mechanisms of periodontitis and discussed how rationally designed bioactive hydrogels can optimize local drug delivery in periodontal therapy, thereby enhancing treatment efficacy and promoting their clinical application [16]. Wang and colleagues summarized the properties of stimulus-responsive hydrogels, which achieve precise drug release in periodontitis by responding to specific microenvironmental cues (e.g., pH, temperature, and enzyme concentrations), thus improving therapeutic outcomes [17]. Pan et al. outlined key hydrogel design principles, including thermosensitivity, self-healing, photocrosslinkability, and adhesiveness, highlighting their multifunctional role as local drug delivery systems with antibacterial, anti-inflammatory, and osteogenic capabilities in periodontitis management, alongside recent clinical trial advances [18]. However, these reviews primarily emphasize hydrogel drug delivery functions, paying limited attention to the unique wet oral environment. Concurrently, some researchers have recognized the significance of the oral cavity's moist milieu; Cheng et al. summarized applications of wet-adhesive materials in repairing diverse oral and maxillofacial soft tissue defects (e.g., mucosal disorders, skin defects, tumors), providing valuable insights into adhesion mechanisms and broad applicability [19]. Jia et al. detailed design strategies for polymers with wet-adhesion capabilities and their multifarious formulations (e.g., nanoparticles, hydrogels, patches, microneedles) in oral disease therapy [20]. Nevertheless, their discussions broadly encompass multiple materials and a wide spectrum of oral diseases, inherently diluting focus on the advantages of wet-adhesive hydrogels and the periodontal pocket's unique microenvironment. Distinctively, our review provides the first systematic and dedicated analysis focusing exclusively on how wet-adhesive hydrogels overcome key challenges posed by the periodontal environment for enabling multidimensional periodontitis treatment.

**Fig. 1:**
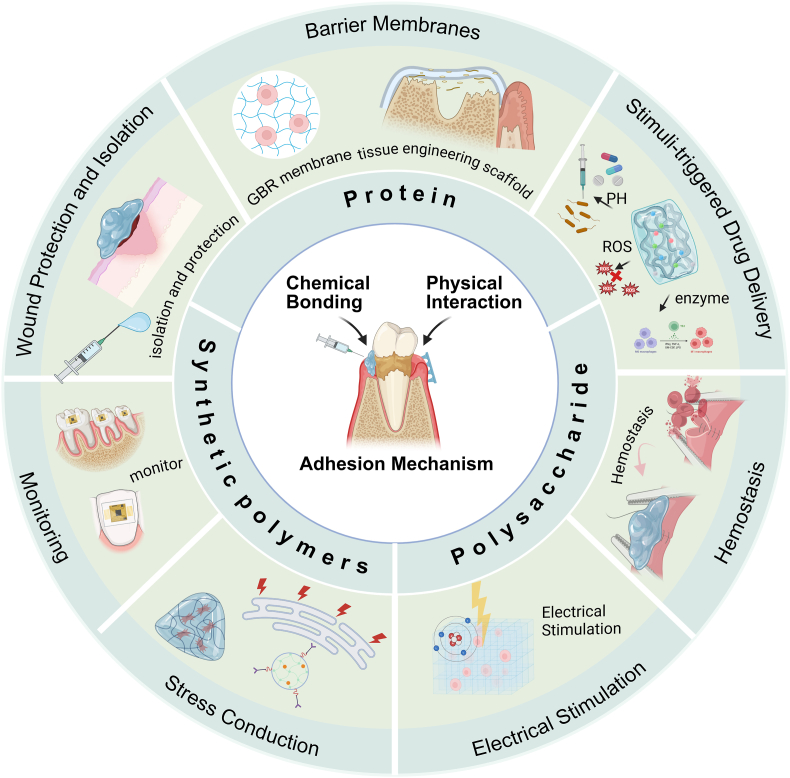
This is a schematic illustration of adhesion mechanisms and multifunctional applications of wet-adhesive hydrogels in periodontitis tissue. (Inner circle) Shows adhesion mechanisms that involve both chemical bonding and physical interactions. (Middle circle) Classifies wet-adhesive hydrogels by material origin: natural polymers (polysaccharides and proteins) and synthetic polymers. (Outer circle) Diverse biological applications encompassing wound protection and isolation, barrier membranes, stimuli-triggered drug delivery, hemostasis, electrical stimulation, stress conduction, and monitoring. (Created by Biorender).

In this review, we focus on recent progress in wet-adhesive hydrogels for periodontitis therapy. We begin by outlining the challenges associated with achieving adhesion in the periodontal environment. This is followed by an overview of various wet-adhesion strategies, highlighting their respective advantages and limitations. Subsequently, we systematically analyze different hydrogel materials, bridging the gap between adhesive mechanisms and clinical applications, and summarizing the diverse applications of wet-adhesive hydrogels in periodontitis treatment. Finally, we discuss the current limitations of wet-adhesive hydrogels and the challenges involved in their clinical translation. Our aim is to provide forward-looking insights into the application of wet-adhesive hydrogels in periodontitis, offering a comprehensive analysis from fundamental mechanisms to clinical translation. (Fig. 1).

## 2 Adhesion challenges

Oral cavity serves as the gateway to both the digestive and respiratory systems [21], presenting a unique environment characterized by salivary pellicle coverage and dynamic functional movements. As the key component of oral mucosa and the primary target site for periodontitis treatment, the gingiva exhibits complex histopathological alterations during disease progression. A thorough understanding of the periodontal tissue's pathophysiological architecture and its complex microenvironment is therefore essential for evaluating the advantages and limitations of hydrogels for periodontal tissues and guiding future directions in wet-adhesive hydrogel design.

Oral mucosa can be classified into three types: masticatory, lining, and specialized [22]. Among these, the gingiva belongs to the masticatory mucosa, which endures pressure and friction during chewing. The gingiva is a critical structure for periodontal health, characterized by its unique pocket-like form. Its inner wall consists of epithelial tissue that normally forms a tight junction with the tooth surface, referred to as junctional epithelium. However, in periodontitis, inflammatory cell infiltration and gingival fluid exudation induce connective tissue degeneration and junctional epithelium destruction, resulting in periodontal pocket formation [23]. Concurrently, the outer pocket wall also exhibits epithelial ulceration and collagen fiber degradation, leading to reduced structural integrity and increased tissue mobility.

Under physiological conditions, saliva and gingival crevicular fluid (GCF) coat periodontal tissue surfaces, providing essential functions like cleansing, protection, antibacterial activity, and digestion [24]. However, these fluids also present a major obstacle to effective hydrogel adhesion and long-term retention. They form a physical barrier that prevents intimate hydrogel-tissue contact, while their continuous secretion and flow create a flushing effect that directly impedes initial attachment and sustained retention [25]. More critically, interfacial fluid molecules compete with tissue surface functional groups for binding sites or contaminate the interface, significantly weakening hydrogel adhesion [26]. In periodontitis, local bleeding introduces blood contamination, which exacerbates both flushing and competitive binding effects, posing a severe challenge to long-term hydrogel retention. Furthermore, dynamic oral activities subject hydrogels to persistent mechanical stress, increasing their susceptibility to detachment or displacement [27]. Collectively, these factors present significant challenges for achieving prolonged, effective local retention of hydrogels.

The pathological microenvironment of periodontitis introduces additional destructive factors that directly compromise hydrogel adhesive stability and material integrity. Microbial metabolism and host inflammatory responses often lower the pH within periodontal pockets to an acidic range (pH 5.4–6.0) [28]. This acidic environment accelerates hydrogel swelling and degradation, reducing overall mechanical strength [29]. Crucially, it also induces protonation of key adhesive functional groups (e.g., amino groups), significantly diminishing their reactivity and capacity to form strong chemical bonds with tissue surfaces [30]. Notably, the pH in the oral cavity/periodontal pocket is not static and may fluctuate dynamically due to dietary intake or salivary buffering [31]. This dynamic instability poses a severe challenge for hydrogels whose adhesion mechanisms or structural stability depend on specific pH conditions.

During periodontitis, the concentration of hydrolytic enzymes, such as matrix metalloproteinases (MMPs), rises significantly within the periodontal pockets. These enzymes specifically recognize and cleave the peptide bonds within natural protein-based hydrogels, thereby compromising their structural integrity [32]. Additionally, the protease gingipains, produced by *Porphyromonas gingivalis*, also contribute to protein degradation. This enzymatic degradation of the hydrogel matrix constitutes a core mechanism leading to adhesion failure. Concurrently, the abundant microbial community in periodontal pockets (e.g., *P. gingivalis*) compromises adhesion not only through protease secretion but also by altering the local microenvironment (e.g., pH, oxygen tension) via metabolic activity and biofilm formation, or by directly eroding hydrogel materials [33]. These actions further impair long-term adhesive stability and functionality. Pathological accumulation of reactive oxygen species (ROS) acts as potent oxidizing agents that attack hydrogel polymer backbones or cleave oxidation-sensitive chemical bonds (e.g., catechol), leading to material degradation and reduced adhesive performance [34].

In summary, hydrogels encounter multiple severe challenges within the complex periodontal microenvironment: interfacial fluids, low pH, enzymatic degradation, microbial activity and ROS, which significantly compromise hydrogel adhesion, accelerate material degradation, and pose critical barriers to sustained retention. A thorough understanding of these limitations is crucial for designing and optimizing next-generation wet-adhesive hydrogel delivery systems for periodontitis.

## 3 Adhesion mechanisms

**Fig. 2:**
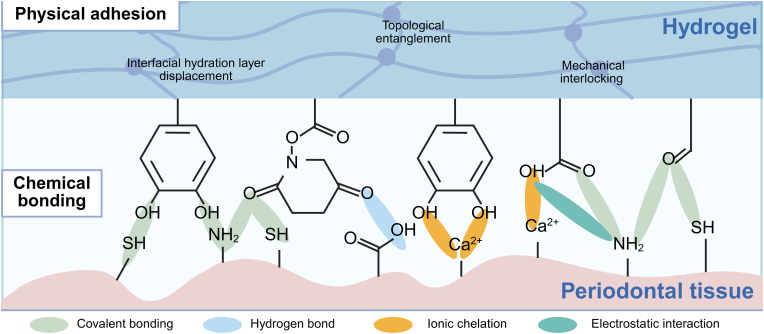
Adhesion mechanisms between wet-adhesive hydrogels and the periodontal tissue, including chemical bonding and physical adhesion.

Periodontal tissues contain various abundant functional groups on their surfaces (such as amino, carboxyl, imidazole, and thiol groups) along with inorganic components (predominantly hydroxyapatite and calcium ions). This composition provides potential adhesion sites for wet-adhesive hydrogels. However, as previously noted, wet-adhesive hydrogels face significant challenges in achieving effective adhesion within the periodontal pocket. This section will critically analyze the potential and limitations of key chemical and physical adhesion mechanisms in light of the characteristic features of the periodontal environment (Fig. 2) (Table 1).

**Table 1:** Advantages and limitations of each adhesion strategy in periodontal environment.

**Strategy**	Chemical reaction (product)	Advantages	Limitations in Periodontal Environment	Improvement Strategies	Ref.
**Catechol**	Covalent: Quinone formation by oxidation, and subsequent Michael addition or Schiff-base reaction; Non-covalent: H-bonding, cation-π, metal coordination	Biomimetic wet adhesion	Slow/pH-sensitive oxidation; ROS generation; Premature oxidation during storage	Enzymatic oxidation (tyrosinase); Antioxidants (ascorbic acid); Boronate ester protection	[35,41,[43], [44], [45], [46], [47], [48], [49], [50]]
**NHS Ester**	Covalent: Aminolysis and thiolysis (Amide bond and thioesters); Non-covalent: Hydrogen bonding	High reactivity	Hydrolysis in saliva/acidic pH	Interfacial water displacement; Combining with other strategies	[34,[51], [52], [53]]
**Aldehyde**	Schiff-base reaction (Imine bonds); Thiol-aldehyde addition reactions (thiohemiacetals)	High reactivity; Dynamic cross-linking (self-healing properties);	Acid-catalyzed bond hydrolysis; Glutaraldehyde toxicity	π-Conjugated aldehydes; Non-toxic alternatives (glyceraldehyde); Optimized crosslinker dose	[[54], [55], [56], [57], [58], [59], [60], [61], [62], [63]]
**Carboxyl**	Ionic chelation; H-bonding/electrostatics; Amidation (amide bonds)	High biocompatibility	Weak wet adhesion; Salivary ion competition	Multivalent cations (Fe^3+^)	[62,[64], [65], [66], [67]]
**Cyanoacrylate**	Anionic polymerization (water or amines)	Instant curing; High bond strength	Rigid interface stress; Exothermic curing process; Degradation byproducts toxicity	Long alkyl chains; Adjunct to sutures	[[68], [69], [70], [71], [72]]
**Interfacial hydration layer displacement**	Hydrophobic water repulsion; Hydrogel water absorption; Dry gel rehydration	Create dry bonding sites; Hemostatic/sealing function	Limited water absorption capacity	Superabsorbent polymers; Hydrophobic monomers (e.g., APA); Xerogel designs	[13,66,[73], [74], [75], [76]]
**Topological Entanglement**	Polymer chain diffusion into tissue matrices	No covalent bonds needed	Influenced by factors such as pH, polymer concentration, and viscosity; Slow adhesion rate	Removing interfacial water; Ultrasound-enhanced penetration	[[77], [78], [79]]
**Mechanical Interlocking**	Photopolymerization (GelMA, PVA-SbQ); Thermo-gelation (PNIPAAm, chitosan); Microneedle	Conventional adhesion; Transdermal drug delivery	Limited light penetration; Microneedle displacement by oral movement	Optimized parameters	[[80], [81], [82], [83], [84], [85], [86], [87], [88]]

### 3.1 Chemical adhesion

**Fig. 3:**
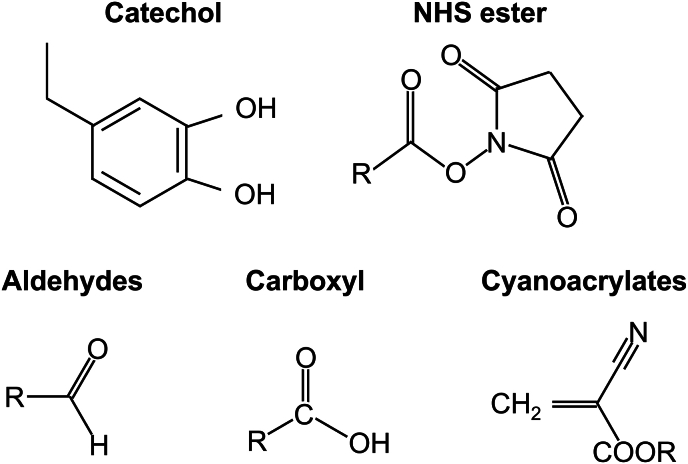
Structural formulas of reactive groups commonly used in wet-adhesive hydrogels for covalent crosslinking with wet tissues.

The majority of existing and emerging wet-adhesive hydrogels rely on specific reactive functional groups to form chemical bonds with the tissue surface. Commonly employed reactive groups include catechol, N-hydroxysuccinimide (NHS) esters, aldehydes, carboxyl, and cyanoacrylates (Fig. 3).

#### 3.1.1 Catechol

**Fig. 4:**
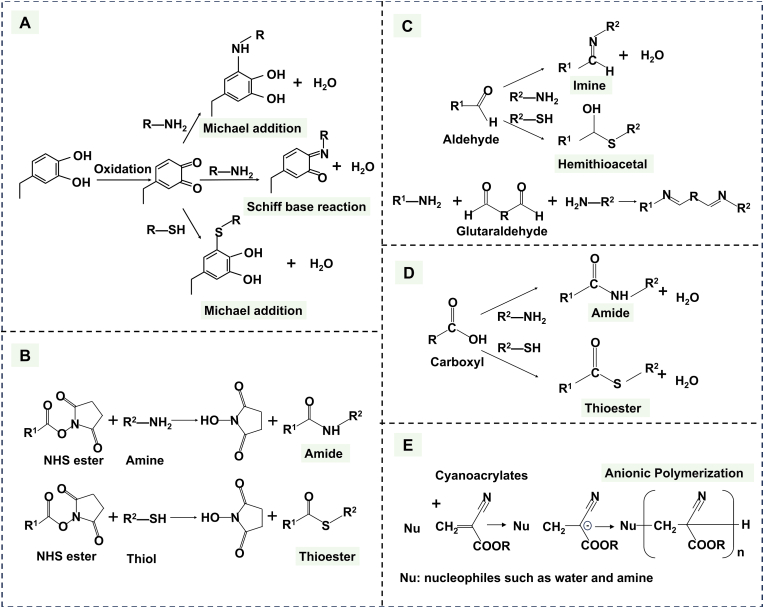
(A) Catechol-mediated adhesion mechanism: Catechol oxidizes to quinone, forming covalent bonds with tissue amines/thiols. (B) NHS ester-mediated adhesion mechanism: NHS esters react with histidine residues and thiols, forming stable amide and thioester bonds respectively. (C) Aldehyde-mediated adhesion mechanism: aldehydes react with histidine/thiols, while glutaraldehyde enables cross-linking via Schiff base formation. (D) Carboxyl-mediated adhesion mechanism: the chemical reactions of carboxyl groups with histamine and thiols involve the formation of amide and thioester bonds, respectively. (E) Cyanoacrylate-mediated adhesion mechanism: cyanoacrylate undergoes anionic polymerization in slightly alkaline conditions, which are initiated by nucleophiles (e.g., H_2_O, amines).

Mussels, known for their exceptional underwater adhesion capabilities, have inspired the development of adhesive hydrogels through the study of their adhesive proteins, particularly mussel foot proteins (Mfps). Among these, 3,4-dihydroxy-L-phenylalanine (DOPA), rich in catechol groups, is considered the most crucial substrate for adhesion [35]. Drawing inspiration from mussels, a diverse library of catechol-based derivatives, such as dopamine, DOPA, norepinephrine, gallic acid, tannic acid (TA) and caffeic acid (CA), has been developed [14,15]. The approach of incorporating catechol groups into polymer backbones to design adhesive hydrogels has garnered considerable interest and been widely investigated for biomedical applications. Commonly utilized polymers include polyvinyl alcohol (PVA) [38], chitosan [39], and sodium alginate [40]. Catechol-mediated adhesion mechanisms typically involve covalent and non-covalent interactions (Fig. 4A).

Catechol can achieve adhesion through reversible non-covalent interactions, including hydrogen bonding, metal complexation, cation-π, and π-π interactions [41]. Drawing inspiration from Mfp-6, thiol groups have been widely adopted to regulate redox balance, thus facilitating durable adhesion [42].

In addition to non-covalent interactions, catechol groups can also be readily oxidized to form semiquinones or benzoquinones in the presence of oxidants, enzymes, or slightly alkaline seawater (high pH). This redox reaction has attracted significant attention in the development of pH-responsive wet adhesives. Quinones can participate in Michael addition reactions or Schiff base reactions with nucleophilic groups, such as primary amines and thiols, forming imine bonds and thiol bonds. These covalent interactions are frequently utilized in the design of adhesive materials for tissue interfaces, such as hydrogel tapes for fault-tolerant strong wet adhesion [21,22]. However, the instability of the quinone structure often leads to self-polymerization and crosslinking via a radical mechanism, which can result in unstable adhesive performance.

Catechol-based hydrogels, despite their biomimetic wet-adhesive potential for periodontal pockets, face significant challenges in achieving efficient adhesion due to their critical dependence on the oxidation state of catechol groups [45], and this is challenging to precisely regulate within the complex periodontal pocket. Catechol's inherent slow self-oxidation tendency creates a dual challenge. Unnecessary oxidation during storage depletes adhesive activity [46]. At the same time, insufficiently rapid oxidation at the target site fails to meet the demands for quick adhesion in the dynamic periodontal pocket. Furthermore, the oxidation process generates ROS [47], potentially exacerbating periodontal tissue damage. Additionally, the low-pH environment within periodontal pockets inhibits catechol oxidation, thereby compromising covalent adhesion, which also presents a significant adhesion challenge [48]. To address these challenges, researchers have adopted strategies. One approach is enzymatic oxidation (e.g., using tyrosinase) for efficient and controlled catalysis that enables rapid covalent crosslinking and robust adhesion [49]. Another strategy involves introducing antioxidants (e.g., ascorbic acid) to scavenge ROS and mitigate tissue damage. Additionally, researchers utilize boronic acid groups to form reversible protective bonds (boronate ester bonds) that prevent premature oxidation and preserve adhesive activity [50]. In summary, the inherent properties of catechol-based hydrogels present fundamental limitations to achieving efficient, rapid, safe, and durable adhesion within the complex microenvironment of periodontal pockets. These unresolved challenges likely explain why the commercialization of catechol-based wet adhesive hydrogels has been hindered.

#### 3.1.2 N-hydroxysuccinimide esters

N-Hydroxysuccinimide (NHS) esters are highly reactive compounds that enable strong adhesion to biological tissues through covalent and non-covalent interactions, making them valuable in wet environments. They rapidly form stable covalent amide bonds and thioesters with primary amines (e.g., in proteins, amino acids, or peptides) and thiols on tissue surfaces (Fig. 4B). Additionally, NHS esters can interact with tissues through non-covalent interactions, such as hydrogen bonding, achieving stable adhesion between materials and biological tissues [34]. In wet environments, although NHS esters are prone to hydrolysis, they preferentially react with primary amines, forming covalent bonds before hydrolysis occurs. As a result, NHS esters are widely used as reactive acylating reagents in wet environments and have broad applications in adhesion [51]. Specifically, within the dynamic periodontal environment, the fast reaction kinetics of NHS esters offer a distinct advantage. However, this setting presents challenges. The constant presence of saliva/GCF and the often acidic pH within periodontal pockets can accelerate the hydrolysis of NHS esters, potentially reducing their bioavailability. Therefore, maximizing the adhesive performance of NHS ester-based systems frequently necessitates combining them with strategies to displace interfacial water [52]. Furthermore, in inflammatory periodontal conditions, additional stability concerns arise. Acidic pH may hydrolyze pre-formed amide bonds, which can actively degrade these covalent linkages. Addressing these degradation pathways is critical for long-term adhesion efficacy. It can be tailored by adjusting reaction conditions (e.g., pH, temperature, and reaction time) to meet specific application needs. Moreover, by ingeniously combining electrostatic interactions, hydrogen bonding, and NHS ester-mediated covalent bonding, hydrogels can achieve rapid, specific, and repeatable underwater adhesion to various biological tissues. This approach enhances adhesion performance while enabling safe removal after treatment and minimizing tissue damage [53].

#### 3.1.3 Aldehydes

Aldehyde groups play a crucial role in the design of functional hydrogels due to their ability to form dynamic covalent bonds. They react with amino groups in tissues to form imine bonds (-N=C-) via Schiff base reactions or with thiol groups to form thiohemiacetals through thiol-aldehyde addition reactions (Fig. 4C). These reactions create reversible, dynamic covalent cross-linking networks that enhance the wet adhesion capabilities of hydrogels and may also impart self-healing properties [54]. The reactions proceed rapidly at room temperature, typically within seconds to minutes. To leverage hydrogel's high reactivity, hydroxyl groups in polysaccharide polymers are often oxidized to aldehyde groups, thereby introducing adhesive properties [55]. Notably, aldehyde-based adhesion is highly pH-dependent [56]. While acidic periodontal environments accelerate imine bond formation, the resulting bonds exhibit inherent instability. To improve stability, π-conjugated structures (e.g., benzene rings) can be incorporated to form benzo-imine bonds [57]. This pH sensitivity is exploitable: Schiff base responsiveness enables design of stimuli-adjustable hydrogels for pH-triggered drug release [58]. Glutaraldehyde serves as a conventional crosslinking agent that stabilizes hydrogel networks while imparting adhesiveness [59]. Despite their adhesive potential, the use of aldehydes often raises concerns regarding cytotoxicity and immunogenicity [60]. Notably, Giu et al. demonstrated that substituting glutaraldehyde with glyceraldehyde significantly enhanced cell viability, suppressed inflammatory factors, and maintained adequate crosslinking density and mechanical properties [61]. Optimizing aldehyde type and concentration thus presents a viable strategy for balancing functionality with biocompatibility [62]. The widespread application of the commercial surgical adhesive BioGlue® (a solution of glutaraldehyde and purified bovine serum albumin (BSA)) demonstrates the enormous potential for clinical translation of aldehyde-based adhesion [63].

#### 3.1.4 Carboxyl

Carboxyl groups interact with the surface of the substrate through physical adsorption mechanisms, such as hydrogen bonding and electrostatic interactions, or through chemical adsorption processes, including the formation of amide bonds via reactions with amine groups and ionic chelation. These interactions increase the contact area and enhance the adhesive strength at the bonding interface (Fig. 4D) [32,33]. In wet environments, the strategic incorporation of hydrophobic properties, combined with drainage mechanisms facilitated by electrostatic interactions and the formation of strong covalent bonds, provides a robust foundation for improving the adhesion of adhesives to moist tissues [62,65]. Natural polysaccharides (e.g., alginate, hyaluronic acid) and synthetic polymers (e.g., polyacrylic acid) contain carboxyl groups that enable electrostatic adhesion in periodontitis pocket. However, adhesion mediated by metal coordination bonds exhibits poor mechanical stability due to its lower bond energy compared to covalent bonds. It is also subject to competition from salivary ions. Coordinated enhancement of cross-linking with multivalent metal ions (e.g., Fe^3+^) is a commonly employed improvement strategy targeting carboxyl groups [64].

#### 3.1.5 Cyanoacrylates

Cyanoacrylate is a synthetic adhesive that cures through anionic polymerization when it comes into contact with water or amines present on tissue surfaces. During polymerization, amines from the tissue can act as Michael-type initiators and become incorporated into the polymer chain, enabling covalent crosslinking between the polycyanoacrylate and the tissue (Fig. 4E). This polymerization reaction occurs extremely rapidly, which is why cyanoacrylate adhesives are commonly referred to as “instant adhesives” or “super glues” [69]. However, their transition from fluid monomers to rigid thermoplastics creates mechanical mismatch with compliant periodontal tissues. In periodontal surgery, the gold standard for post-operative flap closure is suture ligation using silk threads. However, this method can result in biofilm accumulation and tissue trauma. Cyanoacrylates have emerged as valuable adjuncts to traditional suturing techniques, effectively reducing post-operative pain and discomfort while improving flap stability [36,37]. Although studies indicate that commercially available ethyl cyanoacrylate gels/liquids exhibit limited cytotoxicity toward human gingival fibroblasts and osteoblasts [72], their exothermic curing process and degradation byproducts (formaldehyde, cyanoacetate) can induce inflammatory responses, restricting long-term application on hydrated tissues. The curing kinetics of cyanoacrylates are governed by the alkyl chain length in their ester groups. Extending alkyl chain lengths provides a dual-function strategy, which decelerates polymerization (reducing heat generation) while delaying degradation (minimizing toxic byproducts). This structural modification of increasing alkyl chain lengths mitigates cytotoxicity while preserving adhesive functionality [89,90].

### 3.2 Physical adhesion

Chemical adhesion mechanisms enable high-strength hydrogel-tissue bonding through interfacial covalent and non-covalent interactions operating at molecular/atomic scales. Nevertheless, their efficacy is constrained by functional group reactivity, hydrolytic instability in physiological environments, and mismatches in interfacial compatibility. Within the complex periodontal pocket microenvironment—characterized by dynamic hydration, pH fluctuations, and enzymatic activity—sole dependence on chemical bonding often fails to achieve optimal wet adhesion. Here, physical adhesion offers critical complementary advantages.

#### 3.2.1 Interfacial hydration layer displacement

**Fig. 5:**
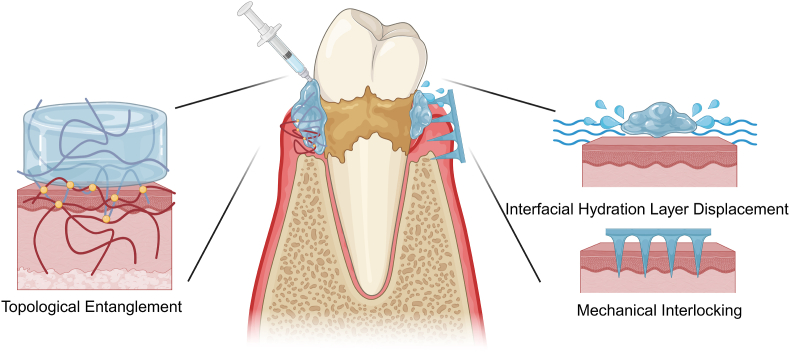
Physical interactions enhancing adhesion: interfacial hydration layer displacement, topological entanglement, and mechanical interlocking. (Created by BioRender).

Effective wet adhesion in liquid environments relies on removing the interfacial hydration layer (Fig. 5) and utilizing adhesion-promoting functional groups. Common strategies include: (1) hydrophobic modification of hydrogels to repel interfacial water prior to tissue contact; (2) hydrophilic components of hydrogels absorbing part of the interfacial water; and (3) pre-formed xerogels absorbing interfacial water to gel in situ.

##### 3.2.1.1 Hydrophobic modification of hydrogels

Hydrophobic modifications enable hydrogels to repel water molecules at the interface, forming a transitional layer between the hydrogel and tissue surfaces. This layer can accommodate only a limited number of water molecules, forcing excess water to move along the interface of the hydrophobic material, thereby removing interfacial water [13]. Rapid and robust adhesion can be achieved through precise regulation of the hydrogel's hydrophobicity, synergistically combined with hydrogen bonding and electrostatic interactions [74]. A notable example is acryloyl phenylalanine (APA), a strongly hydrophobic substance that not only repels interfacial water molecules but also forms hydrogen bonds through its phenyl structure, enhancing both adhesion strength and network stability [75].

##### 3.2.1.2 Hydrophilic properties of hydrogels

Hydrogels consist of three-dimensional (3D) networks formed by highly hydrophilic polymer chains. Functional groups such as -OH, -COOH, -NH_2_, and -SO_3_H attract and retain water, enabling hydrogels to rapidly absorb and retain moisture. While hydrophobic components may not always completely exclude interfacial water, the hydrophilic properties of hydrogels can further remove residual water, achieving close contact and adhesion between the hydrogel and moist tissue [76]. However, this strategy also carries the risk of exceeding absorption capacity limits due to high-flow GCF, which may compromise adhesion durability.

##### 3.2.1.3 Prefabricated dry gels

Due to the limited absorption capacity of hydrophilic gels in their hydrated state, they are often prepared as dry gels. Upon exposure to water, the hydrophilic scaffold rapidly absorbs moisture, instantly forming a gel in situ, which achieves bulk softening and interfacial adhesion. Unlike traditional adhesives, which exhibit indiscriminate adhesion, this material remains non-adhesive and rigid in dry environments but demonstrates induced adhesion and softening upon contact with water. This makes it a promising innovation for applications such as periodontal pocket insertion, where superior adhesion capabilities are required for the treatment of periodontitis [66]. During periodontal inflammation, the periodontal pocket is prone to bleeding and increased exudate. The dry gel can absorb blood and exudate, forming a gel in situ, which not only enhances adhesion but also provides sealing, hemostatic, and reparative functions [73].

#### 3.2.2 Topological entanglement

The topological entanglement between adhesive hydrogels and tissues refers to the complex network structure formed at the molecular scale as polymer chains diffuse and intertwine with tissue surfaces, thereby enhancing the material-tissue interaction (Fig. 5). This mechanism contributes to interfacial adhesion energy independently of specific reactive groups or chemical bonds. A notable example is the rapid infiltration and interconnection of chitosan chains on tissue surfaces, which achieves robust adhesion (>2000 J/m^2^) to wet tissues without forming covalent bonds. The adhesion strength is influenced by factors such as pH, polymer concentration, and viscosity [78]. Other hydrogel polymers that leverage topological entanglement for adhesion include polyacrylic acid, sodium alginate, cyanoacrylates, polyacrylamide, and poly (N-isopropylacrylamide) [79]. One limitation of the topological entanglement adhesion mechanism is its relatively slow adhesion rate, particularly in wet environments. Removing interfacial water to dehydrate the hydrogel matrix can accelerate the interpenetration and entanglement of molecular chains, thereby reducing adhesion time and enhancing adhesion performance. Another potential strategy to accelerate and promote topological entanglement formation is utilizing ultrasonic transducers to induce cavitation and actively embed adhesive polymers into tissues [77].

#### 3.2.3 Mechanical interlocking

Mechanical interlocking enhances adhesion by physically anchoring polymer chains within microscale topographical features of the substrate (e.g., pores, ridges, or surface asperities). This mechanism operates independently of chemical bonding and relies instead on the geometric engagement between the adhesive and the substrate surface. In general, mechanical interlocking improves adhesion by increasing the effective contact area and facilitating additional energy dissipation during crack propagation [81].

##### 3.2.3.1 Mechanical interlocking resulting from filling and curing

For the complex anatomical structures of periodontal pocket, hydrogels must possess excellent fluidity to rapidly conform to irregular shapes, filling every corner and crevice of the periodontal space and achieving intimate contact with the surrounding tissues. Moreover, due to the complex mechanical microenvironment of the periodontium, hydrogels are susceptible to rapid depletion. In-situ solidification, which creates mechanical interlocking, is a commonly employed strategy to extend the retention duration of hydrogels. The primary in situ solidification mechanisms include photopolymerization, temperature-responsive solidification, enzymatic crosslinking, click chemistry reactions, and solvent exchange. Gelatin methacryloyl (GelMA) is the most commonly used photopolymerized hydrogel in periodontitis treatment, relying on the double bonds in its methacrylate (MA) groups to initiate a radical polymerization reaction under ultraviolet or visible light irradiation in the presence of a photoinitiator, thereby forming a covalently crosslinked 3D hydrogel network [82]. Poly (vinyl alcohol) (PVA) hydrogels modified with styrene pyridinium bromide (SbQ) can also form a hydrogel network under ultraviolet light without the need for a photoinitiator, rendering them safer and non-toxic [83]. However, the depth of periodontal pockets limits the widespread application of photo-cured hydrogels. Temperature-responsive solidification is another promising strategy. Thermosensitive polymers, such as Poly (N-isopropylacrylamide) (PNIPAAm), undergo a sol-gel transition near body temperature. This transition occurs when the polymer moves from a liquid state to a gel state due to increased intermolecular interactions, including hydrogen bonding, hydrophobic effects, and electrostatic forces. Polymers with a lower critical solution temperature (LCST) form gels when the ambient temperature rises above this temperature. Conversely, those with an upper critical solution temperature (UCST) form gels when the temperature falls below this temperature [84]. Additionally, chitosan, poloxamer 407 [85], poly (lactic-co-glycolic acid)-poly (ethylene glycol)-poly (lactic-co-glycolic acid) (PLGA-PEG-PLGA) triblock copolymers [86] and poly (D,L-lactic acid)-poly (ethylene glycol)-poly (D,L-lactic acid) (PDLLA-PEG-PDLLA) triblock copolymers [91] are also well-known thermosensitive hydrogels.

##### 3.2.3.2 Mechanical interlocking resulting from microneedle insertion

Conventional adhesive hydrogels primarily rely on direct contact with tissue surfaces, posing challenges of inadequate adhesion in wet environments. As an emerging technology, hydrogel microneedles (Fig. 5) provide a painless and minimally invasive way to penetrate tissue. Notably, their adhesive performance is largely unaffected by wet conditions, offering a simple, efficient, and user-friendly solution for adhesion in challenging environments. Microneedles have been extensively researched for therapeutic applications in recent years [87], particularly for transdermal drug delivery. They enable the maintenance of high local drug concentrations. Importantly, microneedles address the critical challenges of both adhesion and drug delivery in wet environments [88]. Conical-tipped, expandable microneedles undergo deformation upon water contact, which enhances their mechanical interlocking with tissue interfaces. These microneedles achieve adhesion strengths greater than sutures for skin grafts and intestinal tissues, thereby exhibiting immense application potential [80]. The surfaces of these microneedles are designed with micro- or nano-scale structures, such as microprotrusions and microgrooves, which further increase the contact area and friction with tissues, thereby enhancing adhesion.

## 4 The common types of hydrogels

Wet-adhesive hydrogels are extensively utilized in the treatment of periodontitis owing to their strong adhesion and excellent retention capabilities. Both natural and synthetic polymers have been investigated as key components, each imparting diverse physical and chemical characteristics that significantly influence the critical properties of the hydrogels, such as adhesion strength, biocompatibility, rheological behavior, and mechanical performance. In this section, we provide an overview of the types of wet-adhesive hydrogels commonly employed in periodontal applications, aiming to establish a crucial link between adhesion mechanisms and clinical translation (Table 2).

**Table 2:** Summary of hydrogel adhesion performance and key parameters.

Type		Structural Formula	Material System	Adhesion Strength & Test Method/Conditions	Mechanical Durability	Degradation Rate	Biocompatibility	Adhesion Mechanism	Ref.
**Polysaccharides**	Chitosan (CS)		Chitosan-gallic acid graft copolymer (CS-GA)	**27.1**–**41.8 kPa** (Porcine skin lap shear)	Self-healing capability	**40**–**80 %** in 14 days	Cell viability ≈ control group Hemolysis < **10 %**	Chemical adhesion (catechol)	[100]
	Alginate (SA)		SA/Ca^2+^/AANHS/NAGA	**183.6** ± **16.4 kPa** (Porcine skin shear); **275.6** ± **45.0 kPa** (Gingival shear); **298.0** ± **62.1 kPa** (Gingival tensile)	Tensile strength: **205.7** ± **6.9 kPa** Elongation: **245.7** ± **11.4 %** Toughness: **389.7** ± **23.8 kJ m^−3^**	Retention > **120h** in vivo	Cell viability > **80 %** Hemolysis ≈ **1 %** No tissue damage	Mechanical interlocking NHS ester-amine covalent bonding Interfacial dehydration	[110]
			SA/Ca^2+^/Poly (ethylene glycol) diacrylate (PEGDA)	**80 % retention** (5-min underwater flow)	–	–	Cell viability > **95 %** Normal blood/organ histology	Physical (suction)	[101]
			Oxidized SA/Gelatin/Halloysite	Adheres to skin, pork, glass, metal	Self-healing capability Compressive modulus: **332.1 kPa** Stable after 5 × 60 % strain cycles	–	Cell viability > **80 %** Hemolysis < **5 %**	Chemical adhesion (aldehyde)	[105]
	Hyaluronic acid (HA)		Methacrylated HA (HAMA)	–	High strength (100 N compression resistance)	Complete degradation in **90 min** (37 °C) Slowed by NIR	Cell viability > **90 %** No tissue damage	Physical (mechanical interlocking)	[87]
			HA-dopamine (HA-DA)	adhesion to plastics, glass, rubber, and tooth surfaces	Self-healing Injectability	–	Promotes cell migration Hemolysis < **5 %**	Covalent (catechol)	[102]
	Cellulose		Hydroxyethyl cellulose (HEC)	–	–	**33 %** in 48h	No tissue damage	Physical (pocket filling-mechanical interlocking)	[112]
			Oxidized carboxymethyl cellulose (OCMC)/Polyamidoamine Dendrimer Generation 3 (PAMAM-G3)	good adhesivity (original text)	Fracture stress: **9.47 kPa** Strain: **48.69 %** Self-healing	**≈40 %** in 4 days (pH-responsive)	Cell viability > **95 %** Hemolysis < **5 %** No inflammation/toxicity in vivo	Chemical adhesion (aldehyde) Cationic adsorption	[103]
**Proteins**	Gelatin		Methacrylated gelatin (GelMA)/Poly (α-lipoic acid) (PolyLA)	**28.9 kPa** (Oral mucosa) **40.5 kPa** (Porcine skin) table 24 h in dynamic conditions	Dry state: **≈30 MPa** Hydrated: **≈5 MPa**	Degrades in 7d in vivo (rat)	Cell viability > **97.5 %** Hemolysis < **5 %** (GelMA≥25 %)	Hydrogen bonds; Electrostatic interactions; Physical (dehydration)	[66]
	Silk fibroin (SF)		Dopamine-modified silk fibroin (SFD) + chitosan (CS)/β-glycerophosphate sodium (β-GP)	**4.0**–**9.3 kPa** (Porcine gingiva tensile)	Porosity: **45**–**65 %** Swelling: **>80 %**	**50 %** in 14 days (37 °C, artificial saliva)	Cell viability > **95 %** Hemolysis < **1 %**	Chemical adhesion (catechol)	[113]
			Silk fibroin (SF)/gelatin/polydopamine	–	Tensile strength: **25–40 kPa**	Sustained release > **30 days**	Non-toxic; Promotes regeneration	Chemical adhesion (catechol)	[114]
			Silk Fibroin/Tannic Acid/Minocycline Hydrochloride	Blood: Enamel **8.73** ± **1.32 kPa**; Skin **1.25** ± **0.19 kPa** Underwater: Enamel **3.94** ± **0.07 kPa**; Skin **2.32** ± **0.25 kPa**	Self-healing	Complete in **30 days** (protease)	Cell viability > **90 %** Normal cytoskeleton	Chemical adhesion (catechol) Physical (dehydration)	[115]
**Synthetic Polymers**	Poly (vinyl alcohol) (PVA)		Poly (vinyl alcohol)/3,4-dihydroxyphenylalanine/manganese dioxide nanoparticlesPVA/DOPA/MnO_2_ NPs	Holds **500 g** (bovine tooth-gingiva)	Shear-thinning Porous structure	–	No cytotoxicity Low hemolysis No organ damage	Chemical adhesion (catechol)	[38]
			Poly (vinyl alcohol)/Chitosan-coated metronidazole (MTZ) microcapsules PVA@CS@MTZ	**≈15 kPa** (Glass substrate, underwater)	Shear-thinning Injectability	**≈45 %** in 4 weeks	Cell viability > **80 %**	Physical (dehydration) Chemical (H-bonding/amine)	[116]
	Poly (ethylene glycol) (PEG)		Poly (ethylene glycol) diacrylate (PEG-DA)	–	Porosity > **95 %** Swelling; ratio: **36.36** ± **0.18 mg/mg** (24h)	Complete in **18 days** (PBS/α-MEM)	Non-toxic (*in vitro/*in vivo)	Physical (thermogelling/filling)	[117]
			Poly (D,L-lactide)-poly (ethylene glycol)-poly (D,L-lactide) triblock copolymer PDLLA-PEG-PDLLA (PPP)	–	Porous structure	Slow *in vitro* (≈30 days) Degrades in vivo by 4 weeks	Cell viability ≈ control group Low hemolysis No organ damage	Physical (thermogelling/hydrophobic interactions)	[86]
	Polyethyleneimine (PEI)		Oxidized dextran (OD)/Phenylboronic acid-functionalized polyethyleneimine (PBA-PEI)	**4.5 kPa** (Porcine gingiva)	Shear-thinning Injectability	> **90 %** in 14 days (in vivo)	Cell viability > **95 %** Hemolysis < **5 %** No inflammation/toxicity	Covalent (B─N coordination)	[118]

### 4.1 Natural polymers

#### 4.1.1 Polysaccharide

Polysaccharides demonstrate significant anti-inflammatory, antioxidant, and antibacterial properties, making them widely utilized in wound care applications. The polysaccharides discussed in this section include chitosan, sodium alginate, hyaluronic acid, cellulose, and others.

##### 4.1.1.1 Chitosan

Chitosan (CS), the deacetylated derivative of chitin, is the only cationic alkaline polysaccharide found in nature. It is extensively used in the treatment of periodontitis owing to its biocompatibility, biodegradability, antibacterial properties, and hemostatic capabilities [92]. Chitosan's inherent adhesiveness enables strong bonding in humid environments, while its abundant amino groups form amide bonds with tissue interfaces and participate in dynamic covalent bonding. These properties facilitate injectable self-healing hydrogels, which show significant potential for adapting to complex periodontal environments [93]. Furthermore, electrostatic interactions between chitosan's cationic amine groups and anionic phosphate groups on tooth surfaces enhance its adhesive strength [94]. However, the application of chitosan is limited by its poor solubility at physiological pH, weak mechanical properties, uncontrolled degradation rates, and low cell adhesion [95]. To overcome these limitations, researchers have developed a series of chitosan derivatives through chemical modifications, including carboxymethylation [96], sulfation [97], alkoxylation [98], and quaternization [99]. These modifications have enhanced its stability, water solubility, antibacterial properties, and targeted drug delivery capabilities. Feng et al. developed a gallic acid (GA)-modified chitosan hydrogel (CS-GA) that exhibits excellent adhesive properties and can bond to various substrates, including plastic, glass, porcine skin, and bone. The adhesive strength to porcine skin reaches up to 41.8 kPa within 1 h. This remarkable performance is primarily attributed to the catechol structure in GA, which, through the synergistic effects of hydrogen bonding, electrostatic interactions, metal coordination, π-π stacking, cation-π interactions, and covalent bonds, enables the hydrogel to not only exhibit superior adhesive properties but also demonstrate excellent hemostatic and in-situ fixation capabilities in periodontal dressing applications [100].

##### 4.1.1.2 Sodium alginate

Sodium alginate (SA), a natural polysaccharide derived from alginic acid, is primarily extracted from the cell walls of brown algae. Its biocompatibility, biodegradability, high water absorption capacity, low toxicity, and cost-effectiveness make it widely used in medical dressings and drug delivery systems [104]. Despite these advantages, sodium alginate suffers from limitations such as poor mechanical properties and inadequate dimensional stability [105]. These drawbacks are often addressed by chelating its G-blocks with cations (e.g., Ca^2+^, Zn^2+^, Sr^2+^) to form rigid structures or by constructing double-network structures with other polymers (e.g., gelatin [106], sericin [107], chitosan [108]) or incorporating fillers [109]. By adjusting the molecular ratio between the G-blocks of alginate and calcium cations, the stiffness and viscoelasticity of the hydrogel can be precisely controlled.

**Fig. 6:**
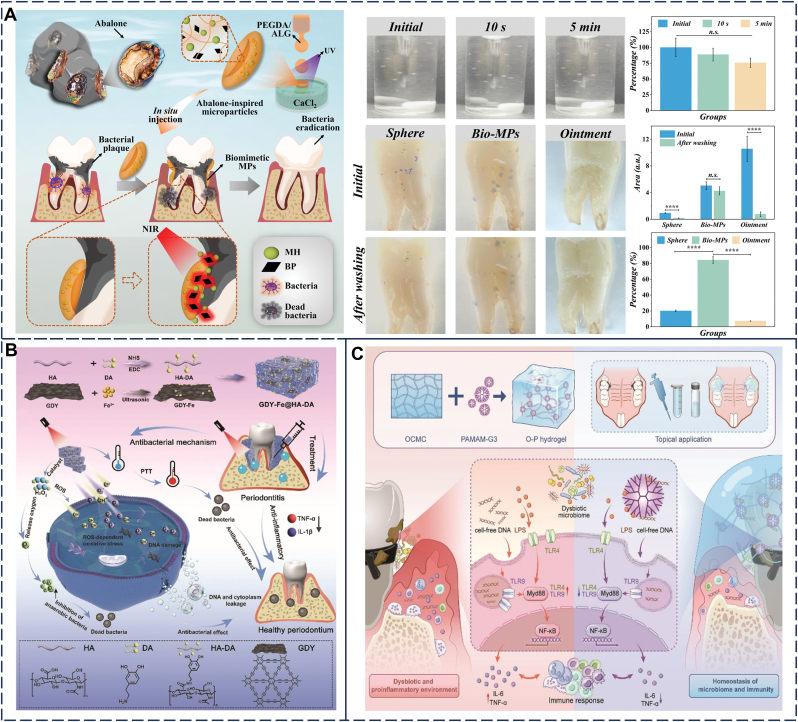
Polysaccharide. (A) Sodium alginate: The graphical illustration of the abalone-inspired microparticles' design, preparation process, and application. Analysis of the adhesive ability of the abalone-inspired MPs. Reproduced with permission from Ref. [101]. (B) Hyaluronic acid: Schematic diagram of GDY-Fe@HA-DA hydrogel synthesis and treatment of periodontitis. Reproduced with permission from Ref. [102]. (C) Cellulose: Schematic illustration of the development of the cationic OCMC-PAMAM-G3 hydrogel and mechanism in periodontitis treatment. Reproduced with permission from Ref. [103].

Based on this strategy, a precursor solution formed by mixing sodium alginate with a low concentration of calcium ions (Ca^2+^) exhibits excellent injectability and moderate viscoelasticity, enabling smooth injection into periodontal tissues and achieving initial retention [110]. In contrast, Song et al. [101] (Fig. 6A) utilized the rapid crosslinking of alginate with a higher concentration of calcium ions to form a stable calcium alginate gel. This gel demonstrates remarkable mechanical strength and stability, capable of maintaining the unique concave structure of the microparticles. With its abalone-like suction cup structure, the gel achieves exceptional underwater adhesion, allowing it to firmly adhere to the surfaces of teeth and periodontal tissues in the wet oral environment.

Notably, sodium alginate faces biodegradation challenges in vivo. As periodontal pockets lack alginate-lysing enzymes, its degradation relies predominantly on gradual divalent cation dissociation. Moreover, degradation products with molecular weights exceeding 50 kDa surpass the renal clearance threshold, raising potential biocompatibility concerns. Oxidation modification has emerged as a critical strategy to mitigate these issues [111]. It reduces molecular weight, accelerates degradation, and introduces new functional groups. During oxidation, carboxyl groups on alginate chains convert to aldehyde groups, creating aldehyde-based adhesives. These aldehydes form highly reactive Schiff bases with amino groups on tissue surfaces, thereby maintaining hydrogel adhesion in periodontal pockets while resolving degradation limitations.

##### 4.1.1.3 Hyaluronic acid

Hyaluronic acid (HA), a natural glycosaminoglycan derived from cartilage and synovial extracellular matrices, can be enzymatically degraded in periodontal pockets by hyaluronidase—unlike sodium alginate. Its degradation products exhibit anti-inflammatory properties and promote neovascularization [119]. It is widely recognized for its biocompatibility, viscoelasticity, water retention capacity, and high swelling ability. However, it also has intrinsic limitations, such as poor mechanical properties, rapid in vivo degradation, and uncontrolled drug release [[120], [121]]. To expand its applications, researchers have developed various chemical modification strategies (e.g., oxidation [82], methacrylation [87]) and crosslinking approaches [122]. These innovative strategies aim to optimize the viscoelasticity, degradation rate, and controlled drug release properties of HA hydrogels, thereby better addressing the requirements for periodontitis treatment. A notable example is the graphene diyne-iron (GDY-Fe) complex delivery platform developed by Wu et al. (Fig. 6B) based on dopamine-modified hyaluronic acid (HA-DA) hydrogels. This nanozyme hydrogel exhibits excellent adhesion properties to a variety of substrates (plastics, glass, rubber, and teeth), primarily attributed to its catechol groups [102].

##### 4.1.1.4 Cellulose

Cellulose, the most abundant polysaccharide globally, is distinguished by its low cost, high abundance, biodegradability, superior functional properties, and ease of synthesis. Nevertheless, its limited solubility in neutral environments restricts its application. Chemically modified cellulose-based hydrogels are produced via targeted chemical modifications to improve stability and water solubility, offering a safer and more effective cellulose-based adhesive hydrogel solution for the treatment of periodontitis [123]. Chen et al. (Fig. 6C) engineered a hydrogel by grafting cationic polyamidoamine dendrimers (PAMAM-G3) onto the backbone of oxidized carboxymethyl cellulose (OCMC) through a controlled chemical grafting process, yielding an injectable cationic hydrogel known as OCMC-PAMAM-G3 (O-P). In contrast to traditional antibiotic treatments, this hydrogel demonstrates the potential to prevent oral dysbiosis and effectively mitigate bone loss, providing a novel therapeutic advantage [103].

Dextran [118] and guar gum have also shown significant potential in periodontitis treatment due to their unique properties and versatile applications. Oxidized dextran, which contains reactive aldehyde groups, forms stable cross-linked networks through Schiff base reactions with chitosan, simplifying the preparation of hydrogel drug delivery systems and enabling efficient drug loading and controlled release [15]. Guar gum, another natural polysaccharide, serves as an effective drug delivery platform due to its biocompatibility and gelation properties [124]. These polysaccharide hydrogels exhibit excellent biocompatibility while allowing intelligent drug release regulation through molecular design, providing precise therapeutic solutions for periodontitis.

Despite the challenging nature of the periodontal pocket, characterized by acidic pH, elevated oxidative stress, and high enzymatic activity, the rich functional group diversity (e.g., hydroxyl, carboxyl, amino, aldehyde groups) of polysaccharide molecular chains represents a significant advantage. These functional groups promote robust intermolecular interactions (such as hydrogen bonding, electrostatic attraction, hydrophobic interactions, and covalent cross-linking), imparting exceptional tissue adhesion within the wet, dynamic, and structurally complex periodontal microenvironment.

#### 4.1.2 Protein

Protein hydrogels demonstrate excellent biocompatibility, biodegradability, and remarkable structural tunability. The proteins discussed in this section include gelatin, silk fibroin, and others.

##### 4.1.2.1 Gelatin

**Fig. 7:**
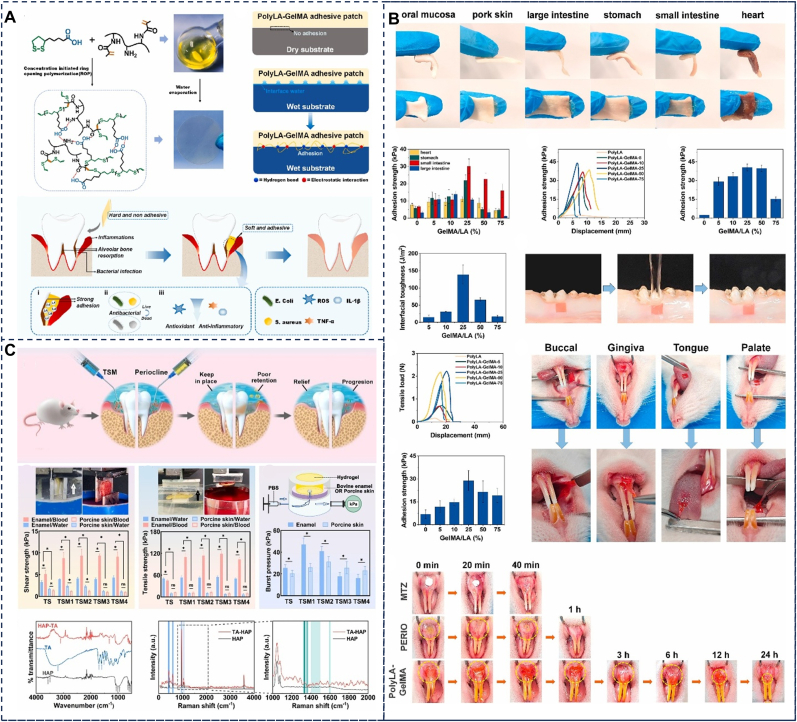
Protein. (A) Gelatin: Schematic diagram of preparation process and structural characteristics of PolyLA-GelMA patch, wet-responsive adhesion behavior of PolyLA-GelMA patch and illustration of the proposed mechanism of PolyLA-GelMA patch for promoting periodontitis healing. (B) Gelatin: Adhesion ability and adhesion strength of PolyLA-GelMA-25 patch to various wet tissues. Reproduced with permission from Ref. [66]. (C) Silk fibroin: Schematic illustration of TSM hydrogels for sustained antibacterial efficacy in periodontitis and the wet adhesion-related properties of TSM hydrogels. Reproduced with permission from Ref. [115].

Gelatin, a protein derived from the controlled hydrolysis of collagen, has been extensively utilized in wound healing due to its excellent biocompatibility, biodegradability, non-immunogenicity, water solubility, and commercial availability [125]. Additionally, gelatin exhibits temperature-controlled reversible phase transition properties, enabling in situ solidification on various biological tissue surfaces and significantly enhancing its adhesion. This characteristic provides significant advantages for the development of localized periodontal delivery systems [126]. However, gelatin-based hydrogels face limitations including low mechanical strength, poor water retention, and rapid degradation by MMPs that are highly expressed in periodontitis [127], which challenges their long-term retention in periodontal pockets. To address these limitations, researchers have developed various gelatin derivatives and modified hydrogels. For instance, gelatin-based hydrogels are combined with crosslinking agents or other polymers to tailor their mechanical properties. Furthermore, moderate physical (e.g., irradiation, high pressure, and ultrasound), chemical (e.g., formaldehyde, aldehydes, and phenols), and enzymatic (e.g., transglutaminase, tyrosinase, and laccase) modifications are employed to enhance the gelatin network structure and functional properties by introducing covalent bonds [128]. Among these, GelMA hydrogels, formed through modification with methacrylic anhydride, have been widely applied in periodontitis treatment [[129], [130]]. For example, Qi et al. designed an elastic patch composed of the coenzyme polymer poly (α-lipoic acid) (PolyLA) and GelMA (Fig. 7A and B), which exhibits excellent biocompatibility and biodegradability for the treatment of periodontitis. Upon contact with the moist environment of the periodontal pocket, the patch spontaneously softens and adheres tightly to the surface of periodontal tissues while releasing the loaded drugs. Additionally, it provides mechanical support to protect the periodontal tissues. The PolyLA-GelMA patch adheres immediately to wet porcine gingival tissue and can withstand vigorous water rinsing without detachment, demonstrating an adhesion strength to oral mucosa of up to 28.9 kPa [66].

##### 4.1.2.2 Silk fibroin

Silk fibroin (SF), an insoluble natural fibrous protein extracted from silkworm cocoons, possesses a hierarchical self-assembly structure that imparts amphiphilicity and the ability to form semicrystalline structures [131]. Stable under physiological conditions, silk fibroin is non-toxic, biocompatible, non-immunogenic, and exhibits excellent mechanical properties, biodegradability, and processability, making it a widely used material in the biomedical field [132]. However, challenges still exist regarding its application in periodontitis therapy. To address these limitations, researchers have enhanced its physicochemical properties through modifications and crosslinking with other polymers [133]. Yang et al. prepared an injectable thermosensitive double-crosslinked hydrogel (SFD/CS) by mixing dopamine-modified silk fibroin (SFD) with the classic thermosensitive hydrogel (chitosan (CS)/β-glycerophosphate (β-GP)), and then crosslinking it with genipin. Due to the presence of catechol, the hydrogel exhibits excellent adhesion, enabling local drug delivery and hemostasis, showing great potential for application in the treatment of periodontitis [113]. Fan et al. (Fig. 7C) designed a wet-adhesive antimicrobial drug delivery system, TA-SF-MCH (TSM), based on a tannic acid (TA) polyphenol modification platform [115]. This hydrogel achieves wet adhesion to different periodontal interfaces by forming coordination bonds between TA's catechol groups and calcium ions in hard tissues, as well as covalent, amide, and hydrogen bonds with the amino groups in the organic components of soft periodontal tissues. TSM-enamel and SM-porcine skin exhibited excellent adhesive strength in both double-distilled water (DD water) and sheep blood, two types of wet environments, within the periodontal microenvironment.

Additionally, sericin [107] and fibrinogen have also been explored for periodontitis treatment. Yin et al. [134] developed an alginate-fibrin hydrogel system encapsulating human periodontal ligament stem cells (hPDLSCs) and metformin, demonstrating significant potential for periodontal tissue regeneration owing to its strong osteogenic properties. These protein-based hydrogels provide innovative solutions for clinical periodontitis treatment.

Taken together, these protein-based hydrogels offer promising biomaterial solutions for periodontitis therapy. Nevertheless, most protein-based hydrogels are derived from natural extraction, which presents challenges including complex extraction procedures, difficulty in purity control, and potential risks of allergies and immunogenicity. These issues urgently need to be addressed for their scaled-up application and clinical translation.

### 4.2 Synthetic polymers

By adjusting parameters such as monomer composition, chain length, and crosslinking density, the porosity, viscosity, elasticity, biodegradability, swelling behavior, and mechanical strength of synthetic polymers can be precisely controlled. This exceptional versatility enables the customization of hydrogel physical properties, making them highly suitable for the treatment of periodontitis. The synthetic polymers discussed in this section include polyvinyl alcohol (PVA), polyethylene glycol (PEG), polyethyleneimine (PEI), polyacrylic acid (PAA), polyethylene oxide (PEO), poloxamer, and others.

#### 4.2.1 Polyvinyl alcohol

**Fig. 8:**
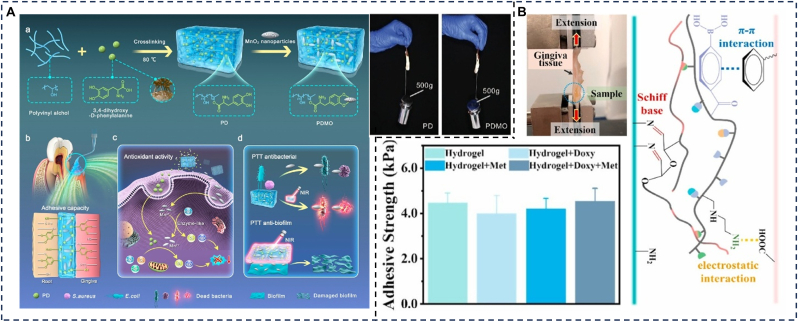
Synthetic polymers. (A) PVA: Schematic illustration of the PDMO hydrogel for treatment of periodontitis and adhesive test of PD and PDMO hydrogel. Reproduced with permission from Ref. [38]. (B) PEI: The adhesion mechanism interpretation of the PBA-PEI/OD hydrogel, representative images of the lap-shear adhesive strength test using pig gingiva tissue and the adhesion strength of the PBA-PEI/OD hydrogels in different groups. Reproduced with permission from Ref. [118].

Polyvinyl alcohol (PVA) is a polymer renowned for its excellent biocompatibility, biodegradability, water absorption capacity, and mechanical properties. It can form a stable matrix structure, maintaining consistent morphology and stability in the oral environment, which facilitates localized drug delivery and clinical manipulation [135]. However, its poor adhesion limits its ability to remain in the moist oral environment for extended periods. To address this limitation and enhance interfacial adhesion, common strategies include incorporating hydrophobic components into the hydrogel to displace interfacial water or constructing double-network systems [136]. For example, Hu et al. [38] developed a mussel-inspired PVA-based hydrogel reinforced with MnO_2_ nanoparticles (NPs), leveraging catechol groups to improve its adhesive properties (Fig. 8A). In adhesion tests performed on bovine incisors and gums, the bonded surfaces could withstand a load of 500 g, demonstrating the hydrogel's ability to remain in the oral environment for prolonged periods despite the presence of saliva and GCF. This hydrogel effectively alleviates periodontitis through sustained antibacterial and antioxidant activities.

#### 4.2.2 Polyethylene glycol

Polyethylene glycol (PEG) is another synthetic polymer with excellent biocompatibility, stability, and hydrophilicity, as well as ease of modification, and it is commonly used as a drug delivery platform for periodontitis [137]. It can be endowed with thermosensitive properties through chemical modification [117]. Wang et al. developed a hydrogel system, PDLLA-PEG-PDLLA, with good thermosensitive switching characteristics. This enables sol-gel transition and enhances its local retention capacity through mechanical interlocking. Degradation experiments demonstrated that the hydrogel rapidly releases stromal cell-derived factor-1 (SDF-1), followed by sustained and stable controlled release of metformin, mimicking the natural bone healing cascade of diabetic periodontal bone regeneration and achieving good results in reshaping osteoblast activity and promoting periodontal bone regeneration [86]. PEG is also frequently used to modify nanoparticles, reducing nonspecific adsorption by forming a surface hydration layer or enabling targeted delivery through further functionalization for the treatment of periodontitis [138]. As an FDA-approved polymer with established non-toxicity, biocompatibility, and low immunogenicity, PEG offers significant advantages for clinical translation in periodontal therapies [139].

#### 4.2.3 Polyethyleneimine

Polyethyleneimine (PEI) is a “comb-like” polymer characterized by a high density of amino groups. Owing to its excellent water solubility, high stability, and tolerance to organic solvents, it is widely used in materials science and drug delivery applications [140]. For instance, Zhao et al. (Fig. 8B) developed a novel injectable localized drug delivery system based on oxidized dextran (OD) and phenylboronic acid-functionalized polyethyleneimine (PBA-PEI). This system achieves robust adhesion through electrostatic interactions, Schiff base formation, and π-π stacking between the hydrogel and tissue. In lap-shear tests using porcine gingiva, adhesive strength reached 4.5 kPa. The hydrogel can be retained in periodontal pockets to enable sustained release of doxycycline and metformin, providing synergistic antibacterial, anti-inflammatory, and osteogenic effects for the treatment of periodontitis [118]. Despite its advantages, the cytotoxicity associated with PEI's high positive surface charge and non-degradable nature may limit its application [141]. To address these issues, strategies such as surface PEGylation, incorporation of degradable crosslinking units, or development of low-molecular-weight derivatives have been proposed to enhance biocompatibility and reduce toxicity.

Other synthetic polymers, such as polyacrylic acid (PAA) [101], polyethylene oxide (PEO) [142], and poloxamer [143], have shown great potential in the treatment of periodontitis. Compared to natural polymers, synthetic polymers offer significant advantages such as lower extraction complexity and absence of inherent immunogenicity, providing favorable conditions for their clinical translation. However, potential biosafety concerns, including cytotoxicity of certain materials or uncertainties regarding long-term in vivo behavior, remain challenges for their widespread application and translation.

## 5 Applications

In recent years, with the rapid advancement of materials science and biotechnology, wet-adhesive hydrogels have demonstrated immense potential in wound protection and isolation, barrier membranes, stimuli-triggered drug delivery, hemostasis, electrical stimulation, stress conduction, and monitoring. For instance, the construction of membranes provides protection and structural support for tissue repair. Through rational design, adhesive hydrogels can not only deliver drugs precisely to deep periodontal tissues but also achieve intelligible release tailored to the pathological microenvironment of periodontitis, enabling synergistic antibacterial, anti-inflammatory, and antioxidant effects. Their exceptional hemostatic properties offer robust control of bleeding in inflamed periodontal tissues. Moreover, adhesive hydrogels can promote osteogenesis through electrical stimulation. Additionally, as a stress conduction platform, they can mimic the mechanical microenvironment of natural periodontal ligaments (PDL) to guide functional tissue regeneration. Even more excitingly, adhesive hydrogels can function as biosensors to monitor the physiological state of periodontal tissues in real time, achieving a breakthrough in integrating treatment guidance with monitoring. This section comprehensively explores the multifaceted applications of wet-adhesive hydrogels in periodontal tissues, aiming to inspire novel ideas and future directions in this field.

### 5.1 Wound protection and isolation

**Fig. 9:**
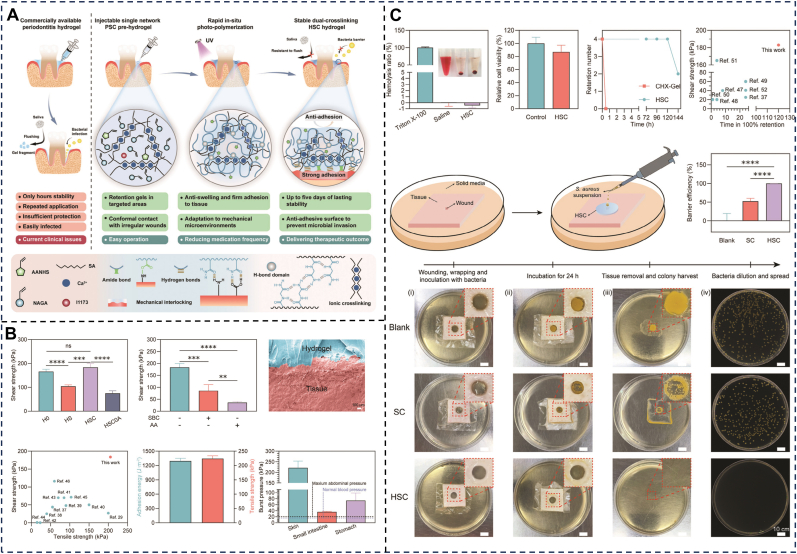
(A) Schematic diagram of commercially available periodontitis hydrogel and in situ light-curing hydrogel for periodontitis treatment. (B) Adhesion properties of hydrogels. (C) Biocompatibility and the long-term protection for the wounds of HSC. Reproduced with permission from Ref. [110].

Periodontitis causes periodontal pocket formation and soft tissue defects. Effective wound coverage not only isolates the complex oral microbiome to reduce infection risks but also maintains an optimal healing microenvironment and provides structural support for tissue regeneration [144]. However, the narrow, irregularly shaped periodontal pocket, which is continuously exposed to the wet and dynamic environment of saliva and GCF, poses significant challenges to the retention of traditional hydrogels. Existing materials often suffer from insufficient adhesion, easy displacement, suboptimal biocompatibility, or performance degradation in wet environments. Wet-adhesive hydrogels, characterized by their excellent hydrophilicity, water retention, biocompatibility, and extracellular matrix-mimetic 3D porous structure, are considered ideal wound dressings [145]. Chen et al. designed an in situ photocurable double-network hydrogel for long-term protection of periodontitis wounds (Fig. 9). The hydrogel precursor is precisely injected into the periodontal pocket and tightly conforms to the surface of periodontal tissues. After UV light curing, the hydrogel forms multiple interfacial interactions—including mechanical interlocking, hydrogen bonds, and amide bonds, achieving strong wet tissue adhesion (shear strength: 275.6 kPa on porcine gingiva; in vivo retention of the hydrogel: >120 h). It also forms a protective barrier that blocks 99.9% of bacterial and harmful microbial entry, reducing infection risks. This study provides a novel strategy for achieving long-term wound sealing and protection in the wet, dynamic periodontal environment [110]. However, mere wound protection and isolation are insufficient for periodontitis treatment. Researchers have incorporated various active ingredients with antibacterial, anti-inflammatory, and antioxidant properties to develop multifunctional hydrogel systems (as discussed in Section 4.4), comprehensively enhancing their protective efficacy and practicality in the complex periodontal environment [146].

### 5.2 Barrier membranes

**Fig. 10:**
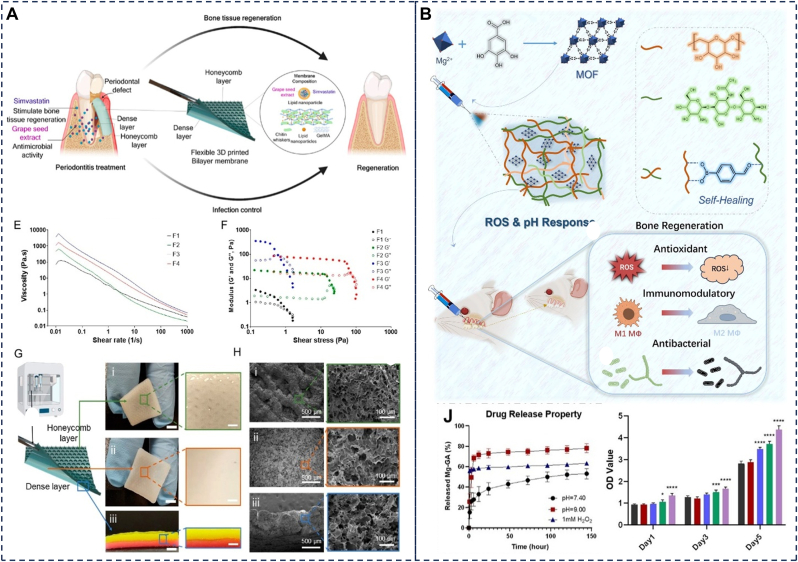
(A) The bi-layered membranes mimic the hierarchical structure of the periodontium, comprising honeycomb and dense layers, characterization of printable composite inks for 3D printing. Reproduced with permission from Ref. [159]. (B) The schematic illustration of the preparation, application and bone regeneration promotion mechanism of CSBDX@MOF and the Mg-GA releasing profiel of CSBDX@10MOF in different conditions. Reproduced with permission from Ref. [161].

Periodontitis frequently leads to bone defects that require guided bone regeneration (GBR). The primary function of GBR membranes is to form a physical barrier preventing soft tissue invasion into osseous defects, creating space for osteogenic cell migration [147]. However, commercially available degradable GBR membranes are often limited by poor space-maintaining capacity, necessitating additional sutures or tacks for fixation, which increases surgical complexity and trauma. Moreover, traditional membranes primarily act as passive barriers and generally lack active immune-regulatory capabilities and osteoinductive activity, limiting bone regeneration efficiency [148]. Adhesive hydrogels, with their plasticity, strong wet adhesion, and biomimetic extracellular matrix properties, have emerged as a promising new generation of GBR materials. They can seamlessly adhere to irregularly shaped bone defect areas and achieve noninvasive fixation [149]. However, their inherent fragility, low mechanical strength, and limited porosity restrict their applications [150]. Strategies such as designing double-network structures [151], incorporating nanofillers [152], adjusting crosslinking density, functionalizing material surfaces, and optimizing preparation techniques (e.g., electrospinning, freeze-drying, and 3D printing) [[153], [154], [155]] are commonly employed to enhance their mechanical properties, porosity, and cellular responsiveness, thereby meeting the mechanical and biological requirements of periodontal tissues. Recent research has focused on developing integrated intelligent scaffolds with “barrier-regeneration-therapeutic” functions [[156], [157], [158]]. For example, Danilo Martins’ team developed a 3D-printed bilayer membrane hydrogel based on hierarchical chitin nanocrystals, exhibiting excellent mechanical properties (Fig. 10A). The dense layer prevents fibrous tissue ingrowth, while the honeycomb layer carries drugs for antibacterial and anti-inflammatory effects. This innovative combination of barrier membranes and regenerative scaffolds provides valuable insights into advanced strategies for periodontitis treatment [159]. Even more groundbreaking is the injectable in situ-forming hydrogel GBR membrane, which precisely fills complex bone defects through minimally invasive injection and possesses both osteogenic induction and antibacterial functions, offering a new paradigm for personalized periodontal regeneration [160].

### 5.3 Stimuli-triggered drug delivery

The pathological microenvironment of periodontitis is characterized by the presence of pathogenic bacterial biofilms and elevated concentrations of ROS [162]. While local targeted drug delivery serves as a core therapeutic strategy, the wet and dynamic oral environment, particularly the morphologically complex periodontal pocket, poses significant challenges to the effective retention and controlled release of drug delivery systems. Conventional non-adhesive systems fail to maintain stable residence within these dynamic pockets and are readily cleared by salivary flow, resulting in subtherapeutic drug concentrations at target sites [18]. Furthermore, their reliance on passive diffusion lacks responsiveness to dynamic pathological changes. Wet-adhesive hydrogels leverage their superior interfacial adhesion to achieve stable retention within hydrated periodontal pockets or bone defects. This provides a robust platform for the targeted delivery of diverse therapeutics (e.g., antibiotics [137], antimicrobial peptides [163], probiotics [164], nanoparticles [165], curcumin [163], CeO_2_ nanozymes [166], glutathione [167]). Critically, the value of these hydrogels extends beyond just passive diffusion. Through precise engineering, they can be endowed with stimuli-responsive properties which trigger on-demand drug release in response to specific pathological cues (e.g., low pH, high ROS levels, or enzymatic activity) [17]. For instance, Luo et al. (Fig. 10B) developed a chitosan-based dynamic hydrogel-MOF (CSBDX@MOF) network exhibiting sensitive responses to ROS and pH. Under simulated pathological conditions (1 mM H_2_O_2_ or pH 9.0), drug release increased significantly, reaching approximately 60% and 74 % within 48 h, respectively [161]. Moreover, release kinetics (e.g., rate, duration, targeting) can be further modulated through infrared irradiation or structural design for optimizing therapeutic efficacy [[18], [168], [169]].

The interfacial anchoring ability of adhesive hydrogels not only addresses the issue of traditional formulations being easily dislodged in the moist oral environment but also enables multi-target synergistic regulation through spatially and temporally controlled drug release. This provides a material foundation for transitioning periodontitis treatment from an “anti-infection” approach to an “immune remodeling” paradigm [170,171].

### 5.4 Hemostasis

**Fig. 11:**
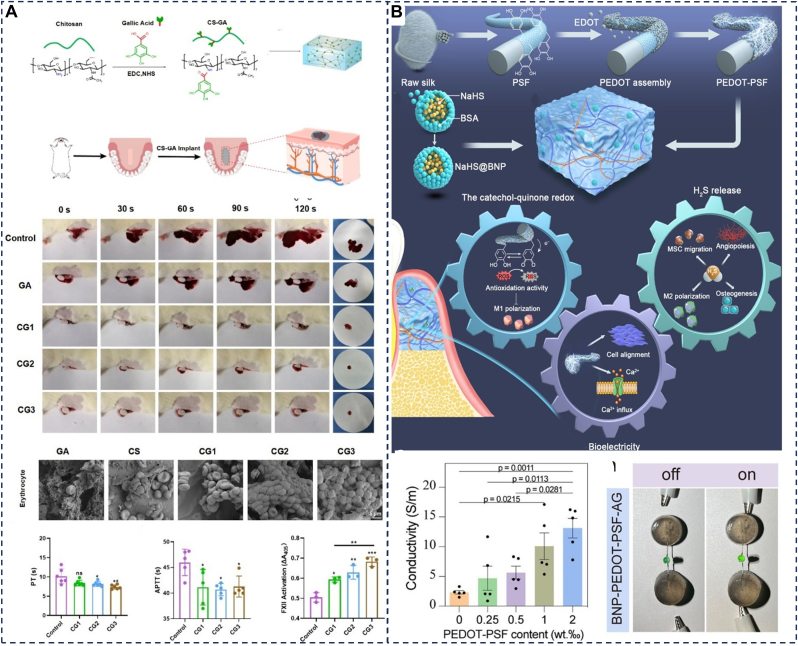
(A) Schematic illustration of CS-GA synthesis and hemostatic performance and mechanism of CS-GA. Reproduced with permission from Ref. [100]. (B) Schematic illustration of the synthesis and properties of the polyphenolmediated redox-active hydrogel with H_2_S gaseous-bioelectric coupling and conductivities of hydrogels. Reproduced with permission from Ref. [106].

Gingival bleeding is a hallmark of active periodontal inflammation [172], and poses significant challenges during surgery, as persistent or profuse bleeding obscures the operative field and hinders the delivery and retention of therapeutic agents. Achieving reliable hemostasis in the wet, dynamic, and morphologically complex oral environment—especially within deep periodontal pockets—remains difficult. Conventional methods (e.g., compression, suturing) exhibit limited efficacy in these confined spaces and the risk of iatrogenic tissue trauma [173]. Adhesive hydrogels, with their excellent wet adhesion capabilities and porous 3D network structures, are increasingly recognized as effective hemostatic materials [113]. They initially adhere to the bleeding site to form a physical barrier that stops blood flow, achieving rapid preliminary hemostasis. Subsequently, their loose and porous structures aggregate and activate red blood cells and platelets, combining physical and biochemical mechanisms to further consolidate the hemostatic effect [174]. The hemostatic efficacy of adhesive hydrogels can be significantly enhanced by incorporating procoagulant components such as coagulation factor VII, thrombin, and calcium ions [[175], [176]]. Feng et al. developed a chitosan-gallic acid graft copolymer (CS-GA) hydrogel, which exhibits excellent hemostatic potential due to its porous structure, high swelling capacity, and strong adhesion (Fig. 11A). By optimizing the grafting amount of gallic acid and crosslinking conditions, they achieved an adhesion strength to 41.8 kPa on pig skin after 1 h of application. Additionally, the hydrogel effectively promotes blood clot formation, which is achieved by adsorbing red blood cells and activating the coagulation cascade, achieving rapid hemostasis within 30 s. Moreover, its antioxidant capacity helps reduce inflammation and oxidative stress, indirectly promoting wound healing. These properties make the CS-GA hydrogel a promising candidate for hemostasis and wound healing in periodontitis treatment [100]. Future work should focus on developing stimuli-responsive, multifunctional hydrogel hemostats that integrate antibacterial, procoagulant, and pro-regenerative functions to address the complex pathologies associated with periodontal lesions.

### 5.5 Electrical stimulation

Electrical stimulation can activate diverse intracellular signaling pathways, influencing the cellular microenvironment, thereby regulating cell migration, proliferation, and differentiation [177]. Substantial evidence supports its therapeutic potential for dental and periodontal tissue regeneration via modulation of oral-derived mesenchymal stem/stromal cells (OMSCs) [178]. However, piezoelectric materials are typically rigid and often lack inherently biocompatible, posing significant challenges for biomedical applications in vivo. By integrating hydrogels with piezoelectric technology, a novel hybrid material system has been developed, combining the electrical stimulation properties of piezoelectric materials with the flexibility, biocompatibility, and biodegradability of hydrogels [179]. When applied in periodontal pockets, this material can utilize biomechanical vibrations from chewing to generate electrical charges, enabling multifunctional effects such as antibacterial activity, osteogenic agent release, and immune modulation [180,181]. Fan et al. developed a gas-bioelectric synergistic piezoelectric system based on a gelatin/sodium alginate hydrogel network. This system incorporates poly (3,4-ethylenedioxythiophene)-assembled polydopamine-mediated silk microfibers (PEDOT-PSF) and a hydrogen sulfide (H_2_S) sustained-release module based on bovine serum albumin nanoparticles (Fig. 11B). This platform synergistically exerts antioxidant, anti-inflammatory, mesenchymal stem cell recruitment, bioelectric release, and immune regulation. In diabetic periodontitis models, it reversed the hyperglycemic inflammatory environment and promoted functional periodontal tissue regeneration [106]. Despite these advances, challenges remain, including relatively low electrical conductivity, potential cytotoxicity of fabrication chemicals, and limitations in fabricating high-resolution conductive polymer architectures. Further optimization of material composition and manufacturing techniques will be essential for translating such bioelectric hydrogel systems into clinical practice.

### 5.6 Stress conduction

**Fig. 12:**
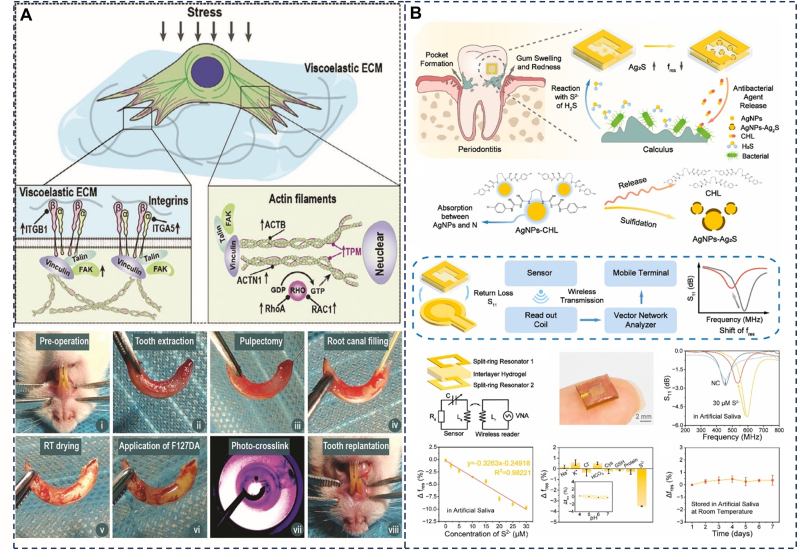
(A) Schematic diagram showing the mechanical regulation of viscoelastic hydrogels on integrin–FAK pathways and the effects of viscoelastic F127DA hydrogels on tooth resorption and PDL healing in the delayed replantation of avulsed teeth in rats. Reproduced with permission from Ref. [186]. (B) The sensing principles and the design of the H2S RF sensors with the read out devices and RF sensors for wireless saliva S2− detection. Reproduced with permission from Ref. [188].

The periodontal ligament (PDL) is a specialized, viscoelastic soft tissue that connects the tooth root to the alveolar bone and confers both shock-absorption and adaptive support under physiological loading [182]. In advanced periodontitis, destruction of the PDL compromises tooth stability and function, making the reconstruction of a mechanically competent, strain-adaptive PDL a central goal of regenerative therapy. The phenomenon of mechanotransduction, where physical forces are converted into biochemical signals that influence cell behavior, serves as the foundation for the role of mechanical cues in promoting wound healing [183]. In the PDL, studies have shown that mechanical forces can promote H-type angiogenesis and osterix-positive (OSX^+^) cell-related osteogenesis, contributing to the maintenance of periodontal homeostasis [184]. Conventional regenerative materials, however, fail to adequately replicate the PDL's complex viscoelasticity or to transmit physiological stress patterns, which limits functional integration and the recovery of normal ligament mechanics in regenerated tissues [185]. Viscoelastic hydrogels overcome these limitations by allowing independent tuning of stress relaxation kinetics and elastic modulus, thereby providing both a mechanically biomimetic environment and efficient stress transfer. Based on this principle, Zhang et al. [186] (Fig. 12A) designed a viscoelastic hydrogel named F127DA specifically for PDL repair. By adjusting the concentration of F127DA, they precisely controlled the hydrogel's stress‐relaxation profile and elastic modulus to that of native PDL tissue. Experiments demonstrated that under dynamic compressive stress (≈0–120 kPa, 1 Hz, 1 h/day), this hydrogel significantly enhanced the production of collagen and fibrosis differentiation-related markers (e.g., COL-1 and SCX) in periodontal ligament stem cells (PDLSCs). Furthermore, the F127DA hydrogel promoted periodontal ligament repair and regeneration in delayed reimplantation experiments using avulsed teeth in rabbits. These findings elucidate for the first time how hydrogels influence cell behavior under mechanical loading and indicate that F127DA hydrogels can utilize mechanical stress to enhance cell-ECM interactions, thereby influencing the fibrosis differentiation of PDLSCs. These findings not only clarify how hydrogel viscoelasticity modulates cell-ECM interactions under mechanical stress, but also establish a proof‐of‐concept for using stress‐conductive biomaterials to guide PDL fibrosis differentiation and tissue regeneration. Looking forward, integrating this stress‐conduction capability with additional functionalities—such as controlled drug delivery or growth factor presentation—represents a promising direction for next-generation, multifunctional periodontal scaffolds [187].

### 5.7 Monitoring

Periodontitis often progresses subclinically, and conventional diagnostic tools, such as periodontal probing and radiographic imaging, are limited by delayed readouts and operator variability, hindering the timely assessment of disease activity. Real-time sensing of inflammatory biomarkers within the periodontal crevice would enable earlier intervention and more personalized treatment regimens [189]. Common biomarkers include sulfides [190], matrix metalloproteinase-8 (MMP-8) [191], interleukins [192], and antimicrobial peptides [193]. Hydrogen sulfide (H_2_S), a metabolite produced by Gram-negative bacteria, is present in high concentrations in the deep periodontal pockets and serves as an important marker for monitoring the disease. Increased H_2_S levels are positively correlated with the severity of periodontitis [194]. The constantly hydrated, dynamic and enzyme-rich environment of the oral cavity poses significant challenges for in situ biosensors, particularly in terms of adhesion stability and signal fidelity. Traditional sensors, limited by inadequate wet adhesion, cannot maintain stable long-term operation for in situ monitoring. Wet-adhesive hydrogels address this limitation through their robust interfacial adhesion, providing an ideal platform for biosensor development. Pan et al. developed a wearable radiofrequency (RF) sensor based on agarose hydrogel, which adheres to the tooth surface (Fig. 12B). The S^2−^ in saliva reacts with silver nanoparticles (AgNPs) to form Ag_2_S, causing a color change in the hydrogel for in situ H_2_S detection and antibacterial treatment. This sensor exhibits high sensitivity, a wide detection range (2–30 μM), and a low detection limit (1.2 μM), enabling accurate differentiation between saliva samples from periodontitis patients and healthy individuals for precise disease monitoring. This hydrogel exhibits a low swelling rate and maintains excellent stability in artificial saliva at room temperature. Simultaneously, the hydrogel loads and releases chlorhexidine (CHL) to treat periodontitis, achieving a breakthrough from single treatment to integrated monitoring and therapy [188]. Despite these advances, several obstacles must be addressed before clinical translation, such as degradation, contamination-induced sensitivity loss, signal instability, biocompatibility, and long-term safety. Addressing these bottlenecks is central to advancing the clinical translation of adhesive hydrogel sensors.

## 6 Clinical translation

Translating wet-adhesive hydrogels into safe and effective periodontal clinical products is a complex, multi-stage process involving material science, biology, engineering, and regulatory science. Tissue adhesives in long-term contact with tissues are typically classified as high-risk (Class III) medical devices by regulatory agencies (such as the U.S. Food and Drug Administration (FDA) and the EU Medical Device Regulation (MDR)) and must undergo rigorous pre-market-approval processes (the FDA's PMA pathway) [195]. Early identification of key translational risks, including material safety, scale-up feasibility, and cost-effectiveness, is crucial for avoiding obstacles and improving success rates. In the following sections, we analyze the current translational status and core challenges of periodontal wet-adhesive hydrogels.

### 6.1 Current status and representative products

Currently available commercial periodontal auxiliary gels, such as Italy's Gengigel® (composed of hyaluronic acid and chlorhexidine) and Japan's Periocline®, have validated the concept of localized drug delivery. However, these products primarily focus on single functionalities, employing passive diffusion for drug release and lacking intelligent responsiveness [115,196]. Furthermore, their wet-adhesive properties are inadequate; for example, Gengigel® instructions recommend that users “avoid eating and drinking for 30 min after application.” Other commercially available oral products, such as Curatick® and Oral Aid®, take the dynamic wet oral environment into account and demonstrate good adhesive properties [197,198]. However, due to their patch form, these products are primarily suited for treating oral ulcers and are not ideal for periodontal pocket anatomies.

These limitations underscore the unmet needs that drive numerous laboratory studies aimed at developing multifunctional wet-adhesive systems specifically tailored to the complex periodontal pocket environment. Notable examples include the mussel-inspired Tannic Acid-Silk Fibroin-Minocycline Hydrochloride (TSM) hydrogel from Sichuan University [115] and the Hydrogel-Sodium Alginate-Ca^2+^ (HSC) photocurable hydrogel from Beijing University of Chemical Technology [110]. By integrating properties such as strong wet adhesion, long-lasting antibacterial effects, anti-inflammatory and antioxidant capabilities, and barrier functions, these advanced materials aim to achieve effective treatments for periodontitis. Promising results have been observed *in vitro* and in animal models. For instance, the TSM hydrogel demonstrated robust underwater adhesion (8.73 ± 1.32 kPa to enamel and 13.55 ± 2.50 kPa to soft tissue) and, in a rat periodontitis model, it reduced the load of P. gingivalis by approximately 50% while suppressing IL-1β expression.

Despite these advancements, most wet-adhesive hydrogels remain in the preclinical research stage. Public database searches (e.g., trialsearch. who.int, using keywords “periodontitis AND adhesion AND gel”) reveal only three instances of periodontal-specific wet-adhesive hydrogels undergoing human trials. Currently, our research group's mussel-mimetic hydrogel is in early-stage trials (*Fabrication of Mussel-Inspired Bioadhesives and Study of Their Clinical Efficacy in Promoting Periodontal Regeneration,* 2025MSXM027). While preclinical data support its therapeutic potential, larger studies are required to confirm its efficacy and safety.

### 6.2 Challenges *in clinical translation*

#### 6.2.1 Material safety and long-term stability

Biocompatibility and non-toxicity at the cellular and systemic levels are fundamental for the clinical use of hydrogels, which also minimize risks of inflammation, infection, and carcinogenicity [199]. For natural polymer hydrogels (e.g., polysaccharide or protein), rigorous purification, sterilization, and validation are essential to address issues related to endotoxins, antigenicity, pathogens, and batch variability, particularly for animal-derived materials [200]. In the case of synthetic polymers, incomplete removal of residual monomers, cross-linking agents, and solvents can pose direct risks to biomedical safety (e.g., glutaraldehyde [201]). Hydrogel properties, including molecular weight, hydrophobicity/hydrophilicity, and charge, also influence biocompatibility; for instance, the high charge density of polyethyleneimine (PEI) can disrupt cell membranes [202].

Long-term stability and controlled degradation are critical factors. Premature degradation can lead to adhesive failures, while excessive retention may impede tissue regeneration or trigger foreign body reactions. It is important to evaluate the clearance and toxicity of degradation byproducts (with renal clearance thresholds around 70 kDa), as excessive molecular weight can increase tissue retention and renal stress, as observed with sodium alginate [203]. Cyanoacrylate-derived formaldehyde has significant in vivo risks [204]. In periodontal applications, the impact of material degradation on microbial balance and long-term safety warrants specific assessment. Potential for local irritation, allergic reactions, and interference with healing in inflammatory environments must also be thoroughly evaluated. These biocompatibility considerations fundamentally guide the selection of materials and components.

#### 6.2.2 Scale-up feasibility

The successful translation of wet-adhesive hydrogels from laboratory concepts to clinically accessible products relies heavily on the feasibility of scalable manufacturing. A critical consideration is whether laboratory synthesis methods can be reliably scaled to meet Good Manufacturing Practice (GMP) standards in terms of efficiency and cost-effectiveness. The complexity of multicomponent formulations or materials sensitive to processing parameters can significantly increase manufacturing difficulty and costs.

#### 6.2.3 Cost-effectiveness

Cost-effectiveness is paramount for successful commercialization in the development of wet-adhesive hydrogels. Total expenses encompass raw materials, labor, manufacturing processes, overhead, and notably, regulatory approval costs. These must be balanced with market potential, which requires addressing unmet clinical needs and demonstrating clear advantages over existing therapies. Ultimately, minimizing production costs is essential to ensure a viable return on investment (ROI).

Despite these challenges, wet-adhesive hydrogel represents a pivotal advancement toward precision, minimally invasive, multifunctional, and intelligent periodontal therapy. Clinical translation extends beyond material performance enhancement, requiring systemic integration of material safety, scale-up feasibility and cost-effectiveness.

## 7 Conclusions

Wet-adhesive hydrogels are rapidly emerging as transformative platforms for periodontitis therapy. Their unique ability to adhere to hydrated tissues provides a robust foundation for developing stable and multifunctional treatments. These hydrogels achieve strong wet adhesion through mechanisms including covalent bonding via diverse adhesive functional groups, hydration layer displacement, topological entanglement, and mechanical interlocking, effectively overcoming the limitations of conventional materials. Additionally, they integrate various functionalities, including wound protection and isolation, barrier membranes, stimuli-triggered drug delivery, hemostasis, electrical stimulation, stress conduction, and monitoring. Collectively, these features demonstrate significant potential for addressing the complex pathological microenvironment and therapeutic needs of periodontitis.

However, the translation of these hydrogels into widespread clinical application confronts formidable challenges. Long-term stability is often compromised within the dynamic physiological environment of the periodontium. There is also a lack of comprehensive clinical validation regarding the optimal balance between multifunctionality and biocompatibility. Furthermore, achieving scalable manufacturing while maintaining cost-effectiveness remains a significant hurdle. These challenges hinder the progression of wet-adhesive hydrogels from laboratory innovations to practical clinical solutions.

To advance this field, near-term priorities should focus on ensuring robust biosafety and long-term stability before moving towards personalized formulations and intelligent systems. The ultimate goal is to develop integrated diagnostic-and-therapeutic platforms. A strong product-market fit is essential for successful commercial translation and adoption. The effective development of bioadhesive technologies relies on careful design considerations related to adhesive performance, long-term stability, functional integration, and biosafety. Continuous interdisciplinary research and collaboration are vital for unlocking the full potential of wet-adhesive hydrogel technology in revolutionizing precision periodontal medicine.

## CRediT authorship contribution statement

**Qinyu Duan:** Writing – review & editing, Writing – original draft, Conceptualization. **Xueyu Wang:** Writing – review & editing. **Shan Wang:** Writing – review & editing. **Zixin Yang:** Writing – review & editing. **Weiwei Tan:** Writing – review & editing. **Zhirui He:** Writing – review & editing. **Shanshan Hu:** Writing – review & editing, Supervision, Project administration, Funding acquisition. **Jinlin Song:** Writing – review & editing, Supervision, Project administration, Funding acquisition, Conceptualization.

## Ethics approval and consent to participate

This is no ethics approval and consent to participant involved in this article.

## Declaration of competing interest

The authors declare that they have no known competing financial interests or personal relationships that could have appeared to influence the work reported in this paper.
